# Discovery of a Highly Selective MC1R Agonists Pentapeptide to Be Used as a Skin Pigmentation Enhancer and with Potential Anti-Aging Properties

**DOI:** 10.3390/ijms20246143

**Published:** 2019-12-05

**Authors:** Eileen Jackson, Marc Heidl, Dominik Imfeld, Laurent Meeus, Rolf Schuetz, Remo Campiche

**Affiliations:** 1 DSM Nutritional Products, Personal Care and Aroma, 4303 Kaiseraugst, Switzerland; eileen.jackson@dsm.com (E.J.); marc.heidl@dsm.com (M.H.); dominik.imfeld@dsm.com (D.I.); rolf.schuetz@dsm.com (R.S.); 2 EuroscreenFast, a Business Unit of EPICS Therapeutics S.A., 6041 Gosselies, Belgium; lmeeus@euroscreenfast.com

**Keywords:** molecular modeling, peptides, skin, MC1R, anti-aging, pigmentation, oxidative stress

## Abstract

One of the first lines of cutaneous defense against photoaging is (a) the synthesis of melanin and (b) the initiation of an oxidative stress response to protect skin against the harmful effects of solar radiation. Safe and selective means to stimulate epidermal pigmentation associated with oxidative stress defense are; however, scarce. Activation of the melanocortin-1 receptor (MC1R) on epidermal melanocytes represents a key step in cutaneous pigmentation initiation and, additionally, it regulates cellular defense mechanisms like oxidative stress and DNA-repair. Thus, making the activation of MC1R an attractive strategy for modulating skin pigmentation and oxidative stress. In this context, we designed and synthesized pentapeptides that act as MC1R agonists. These peptides bound, with high potency, to MC1R and activated cAMP synthesis in CHO cells expressing human MC1R. Using one lead pentapeptide, we could show that this activation of MC1R was specific as testing the activation of other G-protein coupled receptors, including the MC-receptor family, was negative. In vitro efficacy on mouse melanoma cells showed similar potency as for the synthetic MC1R agonist alpha-melanocyte stimulating hormone (NDP-alpha-MSH). Moreover, we could reproduce this activity in human skin tissue culture. The lead pentapeptide was able to induce ex-vivo protein expression of key melanogenesis markers melanocyte inducing transcription factor (MITF), tyrosinase (TYR), and tyrosinase-related protein 1 (TYRP-1). Concerning oxidative stress response, we found that the pentapeptide enhanced the activation of Nrf2 after UVA-irradiation. Our results make this pentapeptide an ideal candidate as a skin pigmentation enhancer that mimics alpha-MSH and may also have anti-photoaging effects on the skin.

## 1. Introduction

Melanocortin-1 receptor (MC1R) is a seven-transmembrane Gs-protein coupled receptor (GPCR) present on the surface of melanocytes [1,2]. MC1R belongs to a family of five melanocortin receptors (MCRs) (MC1R, MC2R, MC3R, MC4R, and MC5R) [3,4,5,6,7,8] which are involved in key biological functions including pigmentation, oxidative stress response, DNA-damage repair, immune responses, feeding behavior, energy homeostasis, sexual function, and sebaceous gland secretion [9,10,11,12]. The natural agonist ligands for the MCRs are derived from proopiomelanocortin (POMC), a polypeptide of 241 amino acids. Four endogenous MCR agonist peptides have been identified including α, β, and γ-melanocyte stimulating hormones (MSHs) and the adrenocorticotropic hormone (ACTH) [13]. These are generated from POMC by proteolytic cleavage by prohormone convertase 1/3 and prohormone convertase 2 [9,10]. alpha-MSH, a tridecaptide, Ac-Ser-Tyr-Ser-Met-Glu-His-Phe-Arg-Trp- Gly-Lys-Pro-Val-NH_2_, binds preferentially to MC1R and is the most important MSH in stimulating melanogenesis and thus cutaneous pigmentation.

Melanin in the skin serves mainly to protect cellular DNA from ultraviolet radiation (UVR)-induced DNA damage [14]. It has a broad absorption spectrum ranging from UV into visible light up to 700 nm [15]. It typically forms supranuclear caps absorbing about 50%–75% of UVR [16] and thus acts as a natural shield against DNA-damage and photoaging. DNA-damage in both keratinocytes, as well as melanocytes, can lead to various cutaneous abnormalities, like actinic keratosis, and in more severe cases, basal as well as squamous cell carcinoma and melanoma [17]. Upon DNA-damage evoked by UVR, alpha-MSH is synthesized and secreted by keratinocytes. It binds to MC1R expressed on the surface of melanocytes to activate the receptor [2]. MC1R is constitutively expressed in human epidermis, but it can be further induced by UVR [18,19,20]. Activation of MC1R sets in motion a signaling cascade through cyclic adenosine monophosphate (cAMP) which leads to the transcription of the genes for melanogenesis. First, the key transcription factor melanocyte inducing transcription factor (MITF) is induced. MITF initiates the transcription of various down-stream targets like tyrosinase (TYR), the rate-limiting enzyme in melanogenesis, and tyrosinase-related protein 1 (TYRP-1), which converts 5,6-dihydroxyindole-2-carboxylic acid (DHICA) into eumelanin [17]. Common pigmentation disorders include vitiligo [21], solar lentigo [22], and age spots [23], which are not only of dermatological concern but also receive ample attention from aestheticians and the cosmetic industry. They have in common that they appear as uneven skin tone. Uneven skin tone is of aesthetic concern in most societies and an even skin tone, be it dark or bright, is preferred [24,25]. The color of the skin is perceived by the human eye as light scattered by chromophores in the skin like, for example, mainly melanin, but also hemoglobin, oxy-hemoglobin, bilirubin, and carotenoids [26]. Thus, a change in one or more of these chromophores affects skin color.

Besides its role in melanogenesis initiation, the alpha-MSH-MC1R system has anti-oxidative properties. It was shown that it can induce nuclear factor erythroid 2 related factor 2 (Nrf2) [27], a key transcription factor in the cellular defense against oxidative stress evoked by UVR [28]. Nrf2 dissociates from its inhibitor Keap1 upon phosphorylation on serine 40, which leads to antioxidant response element mediated transcription [29] of antioxidant and phase II detoxifying enzymes like heme oxygenase-1 [30]. With respect to DNA-damage repair, activation of MC1R by alpha-MSH is positively involved in nucleotide-excision repair [31], and it ameliorated oxidative stress-induced formation of 7,8-dihydro-8-oxyguanine (8-oxodG), a major form of oxidative DNA damage [12]. Hence, the alpha-MSH-MC1R pathway serves as a cellular multiple-line of defense system against the damaging effects of UVR.

Due to the above-mentioned signaling cascades, MC1R is also a target for anti-aging strategies. It is well established that UVR, the main contributor to photoaging, induces oxidative stress, inflammation, and activation of matrix metalloproteases (MMPs) [32,33], which degrade extracellular matrix (ECM) components such as collagens and elastic fibers [34]. This leads to visible signs of aging like wrinkles and skin sagging [35,36,37]. It was found that darkly pigmented skin shows around 10 years delayed signs of photoaging, possibly due to the photo-protective effects of melanin [38,39,40]. In addition, melanocytes are part of a sophisticated and complex skin neuroendocrine network [41]. As such, they secrete neuroendocrine signals essential for cutaneous homeostasis [41,42]. Aging leads to the decline of biochemical and physiological mechanisms also with respect to the skin neuroendocrine system [43]. As such, it was shown that aging affected the expression of MC1R as well as POMC and its derived MC1R-agonist peptides [43,44,45]. Specifically, a marked decrease in MC1R expression was shown in the skin from 60-year-old donors compared to 30-year-old donors, while expression of POMC increased with age in basal keratinocytes but was unaffected in total epidermis [45]. Furthermore, genome-wide association studies found multiple single nucleotide polymorphisms (SNPs) within the MC1R gene and other pigmentation associated genes contributing to perceived facial age [46,47].

The alpha-MSH analog NDP-alpha-MSH [Nle^4^, D-Phe^7^], also known as Melanotan-1 or afamelanotide (MT-I), is a synthetic peptide that induces skin pigmentation [48] and has been approved in Europe for treating erythropoietic protoporphyria (EPP), a skin disease involving phototoxicity which can be ameliorated by inducing skin pigmentation. MT-1 is a universal MCR agonist which can lead to unexpected side effects such as headache and nausea [49].

The search for a smaller more selective and safer peptide sequence that mimics alpha-MSH has been the objective of many research groups [31,50,51]. In addition, the search for molecules with UV-independent pro-pigmenting activity cumulated in the finding of a small salt-inducible kinase inhibitor with melanogenic activity in mice and human skin explants [52].

**Scheme 1:**
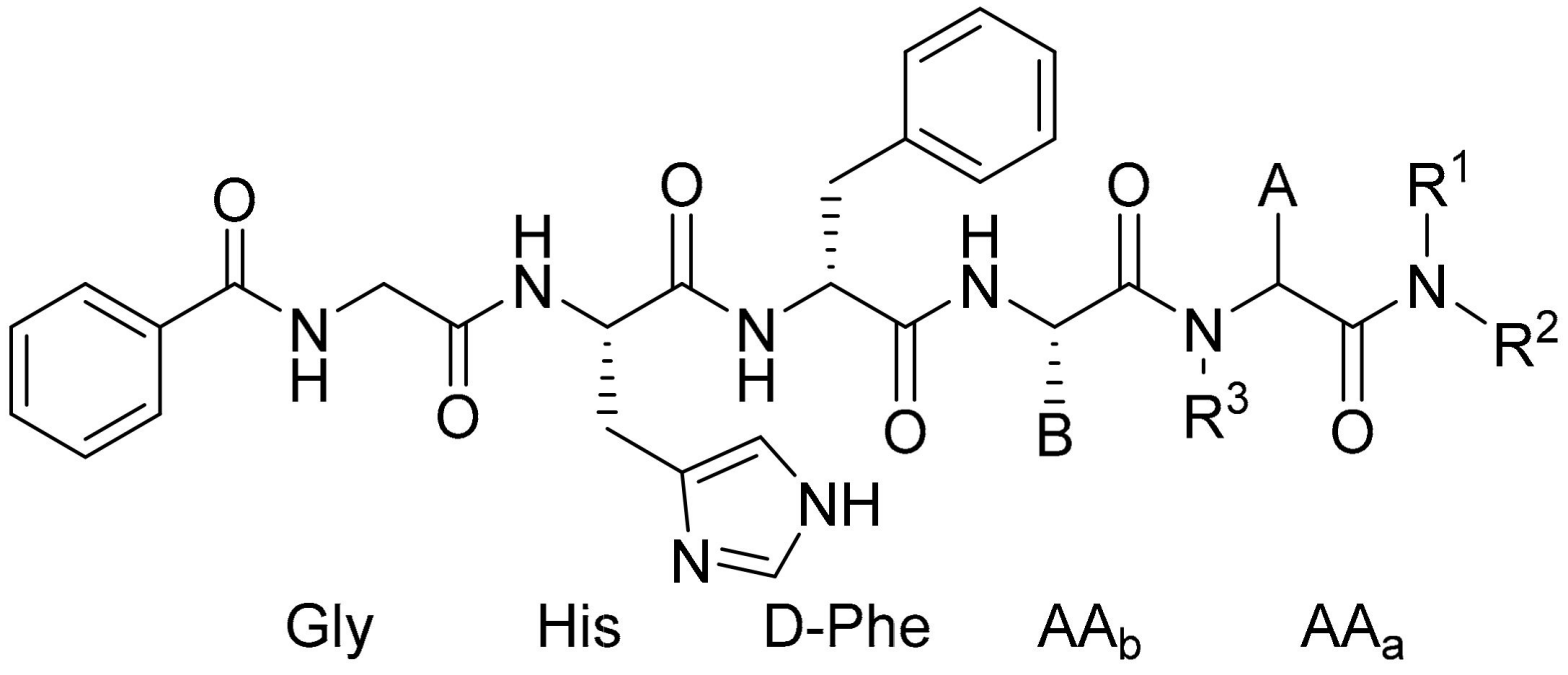
Core peptide sequence.

In this study, we designed and synthesized a novel alpha-MSH-mimicking peptide library. The library was inspired by the His-Phe-Arg-Trp active sequence present in alpha-MSH. The novel pentapeptide sequence features the core sequence Bz-Gly-His-D-Phe-AA_B_-AA_A_-NR_1_R_2_, where AA_B_ is a basic amino acid; AA_A_ is an aromatic amino acid and either amide or alkyl-amide C-terminus (Scheme 1).

Biological testing of this library led to the identification of a novel highly potent and selective MC1R-agonist peptide with alpha-MSH activity making it a promising pro-pigmenting and anti-aging molecule.

## 2. Results

### 2.1. MC1R-Agonist Peptides Activate MC1R

A small library of eight peptides was synthesized and evaluated for their ability to activate the MC1R receptors and induce cAMP production in the recombinant CHO-MC1R cell line using NDP-MSH as control (EC_50_: 0.071 nM) (Table 1). EC_50_ response varied from 0.041 to 7.51 nM.

In terms of the structure–activity relationship (SAR), the most active sequence, 1, was as active as NDP-MSH. Exchange of the *L*-Trp in 1 for a Naphthyl group (2 and 3) also resulted in sub-nanomolar activity, while the incorporation of a *D*-Phe (6) somewhat reduced activity compared to the most active sequence. Exchange of the basic arginine residue for the shorter basic diamino butyric acid, Dab, also decreased the activity. Peptide 7, which features a novel amino acid derivative that mimics Trp, by incorporating the CH_2_-3-indolyl sidechain into an *N*-substituted glycine residue, was also shown to be active, although with a reduced efficacy compared to 1.

### 2.2. MC1R-Agonist Peptides Stimulated Melanin Synthesis in Mouse Melanoma Cells

**Figure 1:**
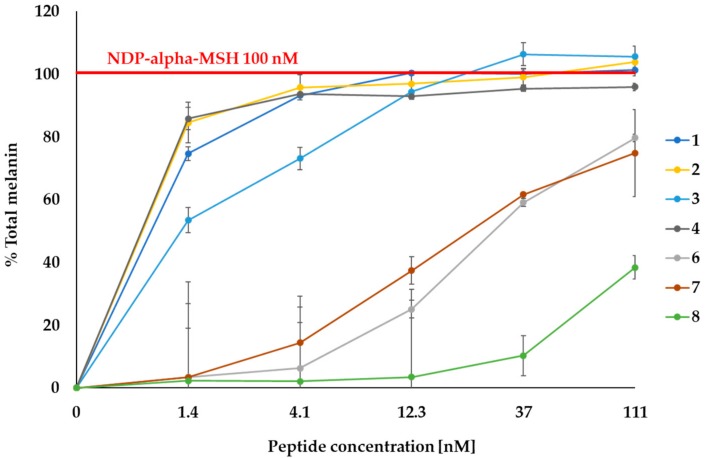
Total melanin synthesis in mouse melanoma cells stimulated with MC1R-agonist peptides. MC1R-agonist peptides were incubated at various concentrations together with the near confluent mouse melanoma cell line B16F10. NDP-alpha-MSH at 100 nM was used as the reference and set to 100% melanin (red line). Error bars represent standard error of the mean of three experiments.

To confirm our binding and activation results on the MC1-receptor in melanocytes, we subjected alpha-MSH-responsive mouse melanoma cells for 72 h to various concentrations of MC1R-agonist peptides (Figure 1). Peptides 1, 2, 3, and 4 reached maximal activity comparable to 100 nM NDP-alpha-MSH at around 12.3 nM. Peptides 6, 8, and 7 showed significantly less activity and reached maximal activity comparable to NDP-alpha-MSH only beyond 111 nM.

### 2.3. MC1R-Agonist Peptides Stimulated Pigmentation in Human Skin Ex Vivo

**Figure 2:**
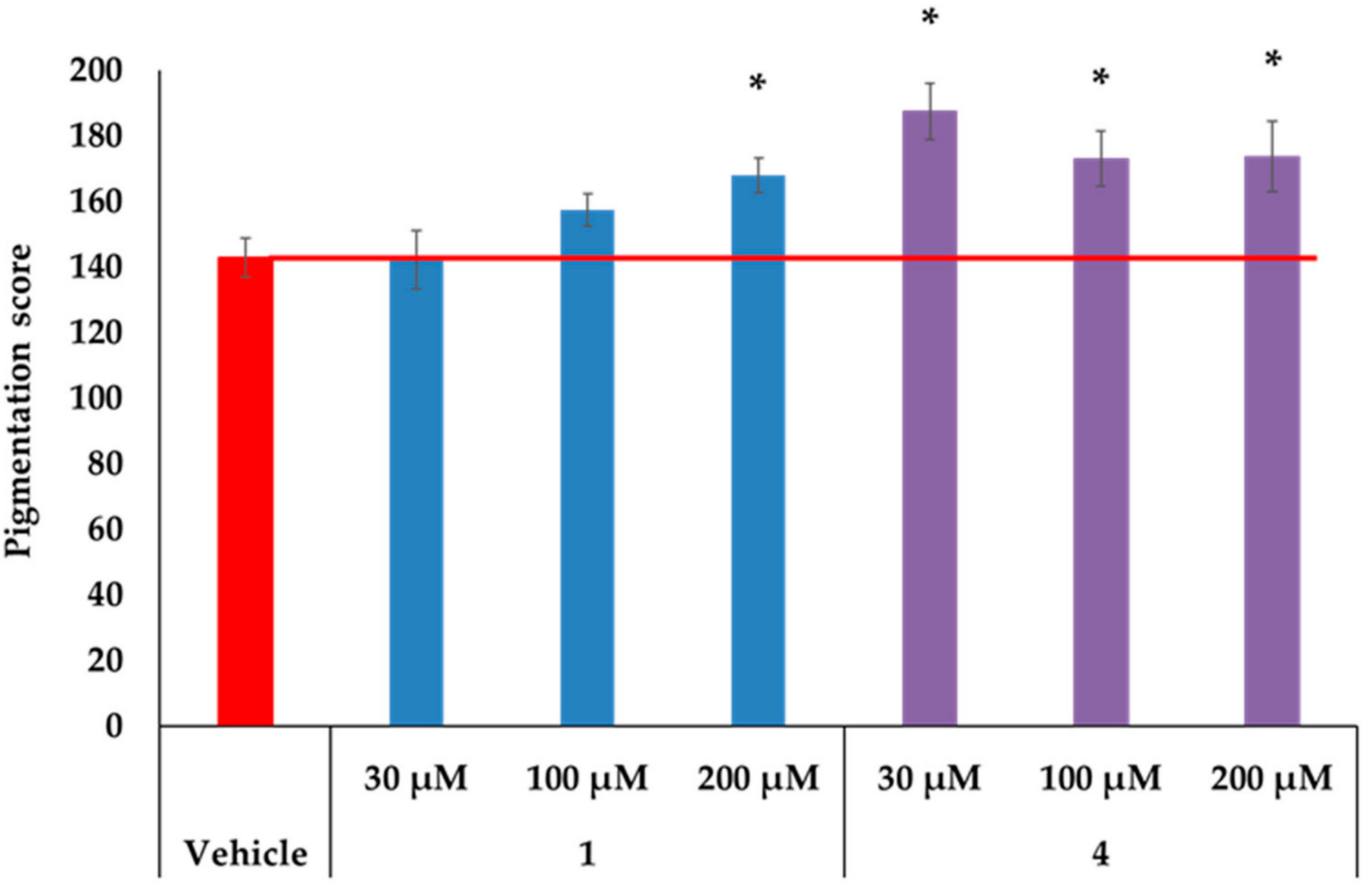
Stimulation of pigmentation by MC1R-agonist peptides ex vivo. Melanin was quantified on skin sections using ImageJ software (NIH, USA). Human abdominal skin was topically treated with peptides at various concentrations for six days. * *p* < 0.05 vs vehicle by unpaired Student’s t-test. Error bars represent standard error of the mean of twelve samples. Red line indicates baseline pigmentation score (vehicle only).

To investigate the melanin synthesis activity of the peptides in a more complex system, we incubated peptides 1 and 4 topically on human abdominal skin ex vivo. Peptides 1 and 4 significantly stimulated pigmentation up to 31% (*p* < 0.05 vs vehicle) compared to vehicle-treated control tissue at 30 µM peptide (Figure 2). Higher concentrations showed a similar activity of up to 21% more pigmentation at 200 µM peptide.

**Figure 3:**
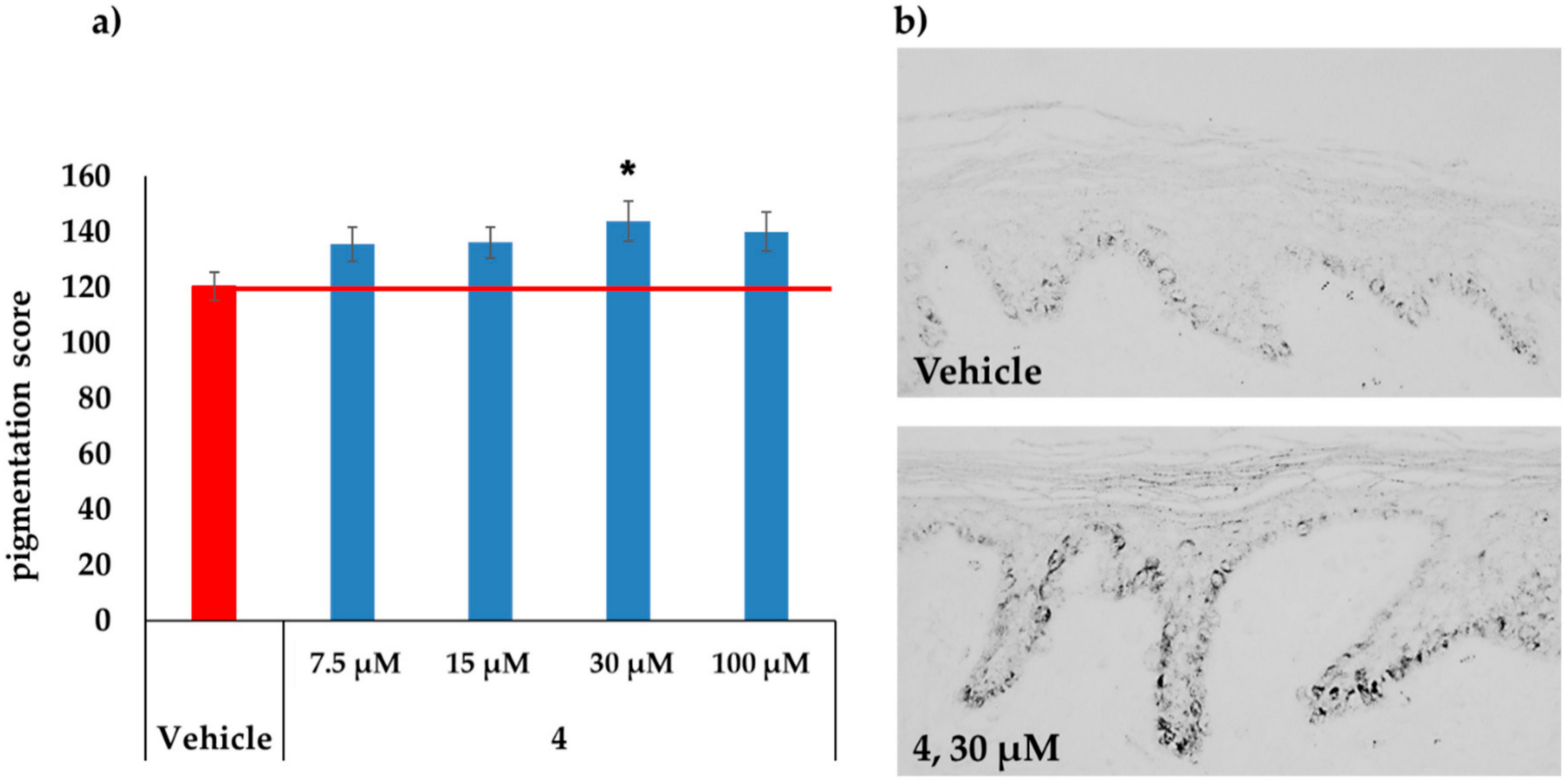
(**a**) The most active peptide ex vivo, 4, had an optimal activity at 30 µM. * *p* < 0.05 by unpaired Student’s t-test. Error bars represent standard error of the mean of twelve samples. (**b**) Representative sections of human abdominal skin. Fontana-Masson staining shows black melanin in melanocytes at the basal membrane. Red line indicates baseline pigmentation score (vehicle only).

We were intrigued by the somewhat reverse dose-dependent activity of peptide 4 and tested it at lower concentrations. This revealed an optimal activity for pigmentation enhancement at 30 µM (Figure 3a). The increase in pigmentation was confirmed using Fontana-Masson staining of human abdominal skin sections treated with peptide 4, where a strong black signal indicative of melanocytes making increased amounts of melanin could be seen at the basal membrane (Figure 3b).

### 2.4. MC1R-Agonist Peptides Activated MC1R Selectively

As peptide entry 4 displayed the highest activity ex vivo, we were interested if its activation of MC1R was specific. To this end, we tested activation of various GPCRs. We selected these GPCRs based on their cellular functions related to MC1R (see Discussion for details). The results of this investigation are shown in Table 2 and they revealed a specific activity of pentapeptide 4 on MC1R with respect to the tested receptors.

### 2.5. MC1R-Agonist Peptide 4 Induced Key Melanogenesis Markers in Human Skin Ex Vivo

**Figure 4:**
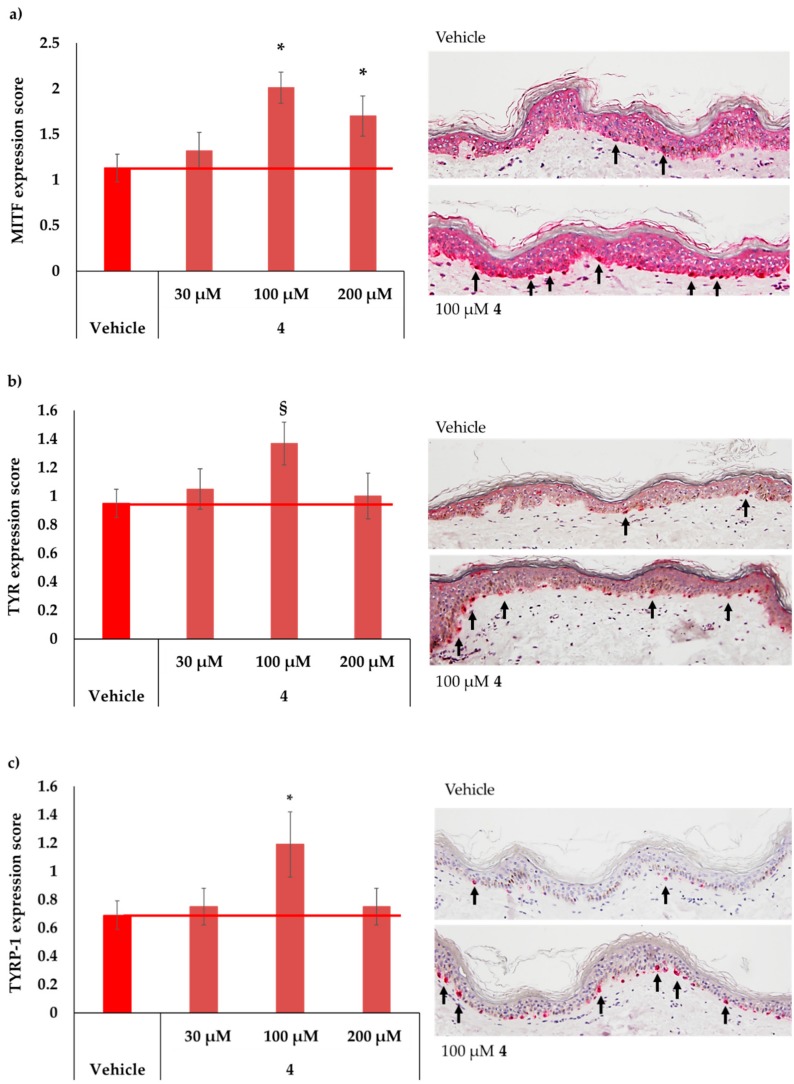
MC1R-agonist peptide 4 induces key melanogenesis markers ex vivo. Skin tissue samples were topically treated with 30, 100, and 200 µM of peptide for six days. Arrows point out red protein immunostaining in basal membrane. Error bars represent standard error of the mean of twelve samples. * *p* < 0.05 and ^§^ *p* = 0.06 vs vehicle by paired Student’s T-test. (**a**) Induction of MITF protein expression is shown. (**b**) Induction of TYR protein expression is shown. (**c**) Induction of TYRP-1 protein expression is shown. Red line indicates baseline protein expression score (vehicle only).

As peptide 4 was able to induce pigmentation in human skin ex vivo, we were interested if it was able to induce the expression of key melanogenesis markers melanocyte-inducing transcription factor (MITF), tyrosinase (TYR), and tyrosinase-related protein-1 (TYRP-1). By immunohistochemistry using antibodies against the three proteins, we could show that indeed peptide 4 was able to significantly induce protein expression of MITF with a maximum of +79% (*p* < 0.05) (Figure 4a). In addition, MITF down-stream targets TYR and TYRP-1 had a maximal induction of +44% (§ = 0.06) (Figure 4b) and +72% (* *p* < 0.05) (Figure 4c), respectively. Interestingly, MITF expression was overall stronger than TYR and TYRP-1 expression.

### 2.6. MC1R-Agonist Peptide 4 Induced Nrf2 Serine 40 Phosphorylation Ex Vivo

**Figure 5:**
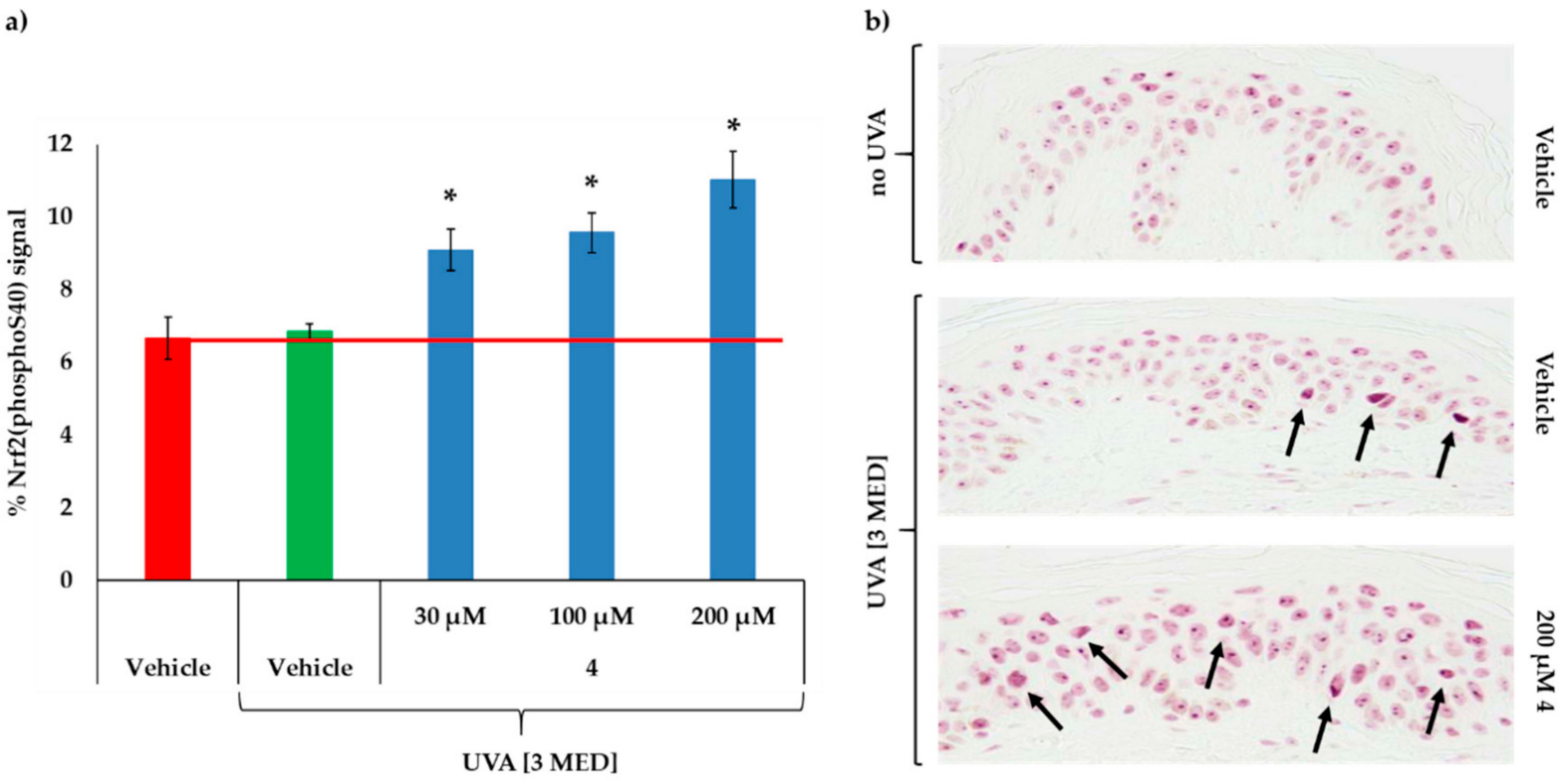
(**a**) MC1R-agonist peptide 4 induces serine 40 phosphorylation of Nrf2 after UVA-irradiation ex vivo in a dose-dependent manner. Error bars represent standard error of the mean. * *p* < 0.05 vs UVA-irradiated vehicle by unpaired Student’s t-test. (**b**) Representative skin sections show immunostaining of Nrf2 (phosphoS40) in nuclei of epidermal cells (arrows). Red line indicates baseline phosphorylation signal (vehicle only).

In addition to the pro-pigmenting effects of peptide 4, we were interested to see if it was able to mimic other known alpha-MSH activities. Alpha-MSH has been shown to induce Nrf2 expression and activity in the presence of UVB [27]. As one important aging mechanism is the generation of oxidative stress after UVA irradiation and Nrf2 is a main transcription factor mitigating UVA-induced oxidative stress [29,30], this could indicate potential anti-aging effects for our peptide. We found that after UVA-irradiation, peptide 4 significantly induced Nrf2 phosphorylation on serine 40 up to 161% (*p* < 0.05 vs UVA-irradiated vehicle control) (Figure 5a), indicative for its transcriptional activation [29]. We found increased nuclear Nrf2 (pS40) staining in UVA-irradiated skin sections of skin treated with peptide 4 (Figure 5b).

## 3. Discussion

In this publication, we describe the discovery of novel MC1R-agonist pentapeptides inspired by the structure of alpha-MSH. The novel peptides were shown to mimic alpha-MSH activity by binding and activating the MC1R receptor in vitro (Table 1), and they were also able to induce melanin synthesis in vitro in mouse melanoma cells (Figure 1). Two of the most active sequences were shown to induce melanin synthesis ex-vivo independent of UVR (Figure 2). Interestingly, the most active peptide in vitro, 1, was not the most active one ex vivo, which could be due to bioavailability issues not present in vitro. As peptide 4 has an alkyl amide group instead of an amide group in 1, which results in higher lipophilicity, it is conceivable it has better skin penetration. Furthermore, the presence of two D-amino acids (D-Phe and D-Trp) as opposed to only D-Phe in 1 may lead to a lower rate of degradation of the peptide by endogenous peptidases. The best performing pentapeptide ex vivo, 4, was further profiled. Protein expression assays confirm that compound 4 activates key melanogenesis markers (TYR, TYRP-1, and MITF) in ex-vivo skin (Figure 4). It was also shown to have an exceptional selectivity profile in a panel of 13 different GPCRs (Table 2). Furthermore, we found that the peptide was able to induce activation of Nrf2 by phosphorylation on Serine 40 upon UVA irradiation ex vivo (Figure 5), hence enhancing the cellular oxidative stress response. Therefore, it may contribute to the skin’s natural shielding mechanism against UVR and help protect the skin against UVR-dependent cellular insults such as oxidative stress. In this way, it can act not only as a pro-pigmenting molecule that contributes to skin tanning and even skin tone, but also as an anti-aging molecule.

We provide evidence that the pentapeptide we selected displays specificity for the MC1R, while it only shows weak potency on MC3R and MC4R (Table 2) and no significant activation of MC2R and MC5R up to 100 µM. In contrast, alpha-MSH has been shown to have a high potency of 5 nM and below among other MCRs [53,54], besides MC1R for which it has the highest affinity. The same was found for NDP-MSH with EC_50_ values below 1 nM. In addition, we tested our functional synthetic alpha-MSH analog on other GPCRs families (Table 2) with related biological activity to MC1R. In this context, melatonin and its receptors, melatonin receptor MT-1 and -2, have been associated with oxidative stress response in skin ex vivo [55]. Furthermore, the beta-endorphin-mu-opioid receptor system was found to be expressed in melanocytes and to be associated with melanogenesis and melanosome formation [56]. Specifically, beta-endorphin is derived from pro-opiomelanocortin and serves as an agonist of the mu-opioid receptor. Concerning the serotonin receptor (5-HT_2A_R), it was shown that serotonin induced melanogenesis in melanoma cell lines through the 5HT_2A_-receptor [57], and the melanin concentrating hormone–melanin concentrating hormone receptor (MCHR) system was suggested to be a functional antagonist of the alpha-MSH-MC1R system [58,59]. Moreover, the endothelin B-receptor, although a Gq-coupled GPCR, unlike MC1R which is a Gs-coupled GPCR, is a stimulator of melanogenesis and acts synergistically with the MC1R pathway [60]. Again, we found that our alpha-MSH analog showed no measurable activity on these GPCRs (Table 2), suggesting specificity of peptide 4 for MC1R.

Concerning biological activity, the peptide repeatedly showed optimal activity at around 30 to 100 µM. For example, pigmentation of ex vivo skin was highest at 30 µM and slightly decreased at 100 and 200 µM (Figure 2 and Figure 3a), while still being present. This suggests a tanning activity in human skin in vivo. A comparison to other already developed alpha-MSH peptide analogs is difficult, as for most of them no dose-dependent cellular activity is given [61], or they were tested in melanocytes in vitro [31]. A recent report showed a dose-dependent increase of melanin synthesis in mouse melanoma cells by alpha-MSH reaching a plateau at around 1 to 10 nM [62]. Interestingly, it was found that POMC-derived peptides, such as alpha-MSH and beta-endorphin, could modulate the expression of their respective receptors [45,63]. Thus, we could hypothesize that as we increase the alpha-MSH signal using the pentapeptide, we at the same time decrease the receptor leading to an attenuation of melanin synthesis. However, we found that the pentapeptide had no influence on the expression of MC1R in ex vivo epidermis in the presence of UVA (Figure S1), contradicting this hypothesis. While we cannot at this point fully explain the occurrence of this decrease in pigmentation at higher concentrations ex vivo, we propose this to be a putative safe mechanism preventing the peptide from contributing to hyperpigmentation and possible melanoma. Such had been suggested to be the consequence of lack of melanin removal at sites of chronic sun exposure [23]. While alpha-MSH through MC1R not only activates pigmentation but also other signaling pathways, we investigated a possible role for our pentapeptide in oxidative stress response. For example, it had been shown that UVR led to the formation of hydrogen peroxide decreasing the expression of catalase and the generation of 7,8-dihydro-8-oxyguanine (8-oxodG), which could be mitigated by pre-incubation of cells with alpha-MSH [12]. We found increased phosphorylation of Nrf2 at serine 40 suggesting nuclear translocation and activation of the protein. This is in line with previous findings that alpha-MSH induced expression of Nrf2 and downstream genes, like heme-oxygenase and glutathione-S-transferase, both in keratinocytes and melanocytes in vitro [27]. Mitigation of UVR-induced oxidative stress and DNA-damage was also shown for a range of alpha-MSH mimicking tripeptides in vitro in melanocytes, further supporting our findings in ex vivo skin [31]. The alpha-MSH-MC1R signaling system has many diverse biological effects. Among these described effects are, for example, cutaneous wound healing [64], effects on the immune system [65] or nucleotide excision repair [31,66], but these were not investigated in this study.

Furthermore, as the pentapeptide described here is an activator of MC1R, its activity likely depends on the presence of a functional receptor. Up to about 200 genetic variants of MC1R are known. These comprise full or partial loss-of-function variants [67], as these variants show decreased MC1R signaling and thus decreased synthesis of eumelanin. Usually they are associated with red hair and increased melanoma risk due to the predominant presence of pheomelanin instead of eumelanin [65,67]. In this respect, we hypothesize that our pentapeptide needs a functional MC1R to stimulate pigmentation and anti-aging effect, such as anti-oxidant activity in skin. We only tested our peptide in skin explants from individuals with an intermediate to tanned phenotype [68,69] for pigmentation effects and in explants from a skin type II [70] individuals for the stimulation of Nrf2. Therefore, further studies would be needed in order to ascertain the effects of our peptide in individuals with MC1R mutations.

In summary, we report the development of a novel synthetic pentapeptide mimicking alpha-MSH with melanogenic and anti-oxidative activity in the skin.

## 4. Material and Methods

### 4.1. Synthesis

Entries 1–3 and 6–8 were prepared by straight forward solid phase synthesis on a Rink amide resin. After stepwise solid phase synthesis, all protecting groups present were removed and the peptide was simultaneously cleaved from the resin. Subsequently the peptides were purified by preparative HPLC (Waters Corporation, Milford, MA, USA).

Entries 4 and 5 were prepared by straight forward solid phase synthesis on a 2-Chlorotritylchloride resin. After stepwise solid phase synthesis, the fully protected peptide was cleaved from the resin. After a carefully controlled fragment coupling, all protecting groups present were removed and the peptide was purified by preparative HPLC. Synthesis and characterization of entries 1–8 are described in detail in Supplementary Materials.

### 4.2. MCR-Activation Assays

MCR-activation assays were performed by means of a cAMP HTRF (homogeneous time resolved fluorescence) assay for Gs-coupled receptors (cAMP Gs dynamic kit, Cisbio bioassays, 62AM4PEC). CHO-K1 cells expressing human recombinant MC1 receptor (EuroscreenFast, Gosselies, Belgium) (or MC2R, MC3R, MC4R, MC5R) were grown prior to the test in media without antibiotic. They were detached by gentle flushing with PBS-EDTA (5 mM EDTA), recovered by centrifugation, and resuspended in assay buffer (5 mM KCl, 1.25 mM MgSO_4_, 124 mM NaCl, 25 mM HEPES, 13.3 mM Glucose, 1.25 mM KH_2_PO_4_, 1.45 mM CaCl_2_, 0.5 g/L BSA, supplemented with 1 mM IBMX). Dose response curves were performed in parallel with the reference compounds. For agonist test (96-well), 12 µL of cells were mixed with 12 µL of the test compound diluted at increasing concentrations in assay buffer, and then incubated for 30 min at room temperature. After addition of the lysis buffer and 1 h incubation, fluorescence signal was measured, according to the manufacturer’s specifications, with the homogeneous time resolved fluorescence (HTRF) kit [71].

### 4.3. MT- and OP-Receptor Activation Assays

Melatonin (MT1R and MT2R) and opioid receptor (OP1R, OP2R, and OP3R, corresponding to human delta, kappa, and mu opioid receptors) activation assays were performed by means of GTPγS scintillation proximity assays. For agonist testing, membrane preparations from CHO-K1 cells expressing each receptor (EuroscreenFast, Gosselies, Belgium) were mixed with guanosine diphosphate (GDP) (*v/v*). In parallel, GTPγ[^35^S] (PerkinElmer, NEG030X, 0.1 nM final assay concentration) was mixed with PVT-WGA beads (Perkin Elmer, RPNQ001, 0.5 mg/well final assay density) (*v/v*) just before starting the reaction. The following reagents were then successively added in the wells of an Optiplate (Perkin Elmer): A total of 50 µL of test or reference ligand diluted in assay buffer, 10 µL of assay buffer (20 mM HEPES pH 7.4; 100–200 mM NaCl, 10 µg/mL saponin, MgCl_2_ at optimized concentration for the specific receptor, 0%–0.1% BSA), 20 µL of the membranes: GDP mix, and 20 µL of the GTPγ[^35^S]: Beads mix. The plates were covered with a top seal, mixed on an orbital shaker for 2 min, and then incubated for 1 h at room temperature. Then the plates were centrifuged for 10 min at 2000 rpm, incubated at room temperature for 1 h, and counted for 1 min/well with a PerkinElmer TopCount™ reader.

### 4.4. MCH-, ETB-, and 5-HT_2A_-Receptor Activation Assays

Melanin-concentrating hormone receptor (MCHR), endothelin-1 receptor (ETBR), and serotonin-receptor (5-HT_2A_R) assays were performed by means of Aequorin assays [72]. Recombinant cells co-expressing human MCH1R, MCH2R, ETBR, or 5-HT_2A_R and mitochondrial apoaequorin (MCH1R, MCH2R and 5-HT_2A_R cells also co-express recombinant Gα_16_ protein) (EuroscreenFast, Gosselies, Belgium), grown 18 h prior to the test in media without antibiotics, were detached by gentle flushing with PBS-EDTA (5 mM EDTA), recovered by centrifugation, and resuspended in assay buffer (DMEM/HAM’s F12 with HEPES + 0.1% BSA protease free). Cells were incubated under agitation at room temperature for at least 4 h with Coelenterazine h (Promega, X300X, 0.5 µM final concentration). Dose response curves with the reference compounds were performed before testing the compounds. For agonist testing, 50 µL of cell suspension was injected on 50 µL of test compound or reference agonist diluted in assay buffer and plated in a 96-well plate. The resulting emission of light was recorded using the Hamamatsu Functional Drug Screening System 6000 (FDSS 6000). To standardize the emission of recorded light (determination of the “100% signal”) across plates and across different experiments, some of the wells contained the reference agonist at a concentration equivalent to the EC_100_. Agonist activity of test compound was expressed as a percentage of the activity of the reference agonist at its EC_100_ concentration.

### 4.5. Cell Culture for Melanogenesis Stimulation

B16 murine melanoma cells, which are highly responsive towards stimulation with alpha-melanocyte stimulating hormone (alpha-MSH), were grown in Dulbecco’s modified eagle medium (DMEM) with 1 g/L glucose supplemented with 3 g/L glucose, 2 mM l-glutamine, 50 U/mL Penicillin, 50 µg/mL Streptomycin, and 10% fetal calf serum. They were kept at 37 °C in a 5% CO_2_ atmosphere. Cells were grown for 24 h and then medium was replaced with medium containing the test or reference (NDP-alpha-MSH) compounds. After 72 h, total (intracellular and extracellular) melanin content was determined by absorbance spectroscopy at 405 nm. The results were quantified against a melanin standard curve.

### 4.6. Tissue Culture to Assess Induction of Melanogenesis

Following declaration of Helsinki principles (DoH-Oct2013, World Medical Association, Ferney-Voltaire, France), human skin samples were obtained from elective plastic surgery after informed consent was obtained. For the two experiments shown in Figure 2, Figure 3 and Figure 4, we had one female donor age 47 with an individual topology angle (ITA°) of 18, and one male donor age 63 with an ITA° of 39 [69]. The skin samples were cut in pieces of 8 × 3 mm and cultured up to day 6. Skin samples were cultured in an air–liquid interface in a perforated ring of stainless steel in contact with culture medium (modified Williams’ E medium). The culture medium was renewed every three days (i.e., day 0–3). Compounds were added daily in DMSO at 4 µL volume. At day 6, the skin samples were fixed in formalin, embedded in paraplast, and cut at the microtome for histochemical staining and consequent image analysis. Six skin biopsies were used for each single treatment.

Two skin biopsies for each treatment were weighed and, in each case, reduced in the dermal portion, in order to have approximately the same weight for all of them. Samples were then washed and processed with methylthiazolyldiphenyl-tetrazolium bromide (MTT) [73]. The color intensity, that is directly proportional to skin vitality, was measured with a plate reader at a wavelength of 570 nm. For melanin quantification, skin sections were stained with a modified Fontana-Masson stain that dyes melanin argentaffin granules in black/dark grey. The amount of melanin present in each slide was evaluated by estimating grey intensity and distribution within the sections using ImageJ application (NIH-USA). Two slides of each skin sample were processed by image acquisition and related analysis (i.e., 12 images for each treatment).

### 4.7. Immunohistochemistry of Melanogenesis Markers

Twelve skin sections from the ex vivo melanogenesis experiment above were immunoassayed with the selected antibodies (TYR, Thermo Scientific #MS-800-P0 (Thermo Scientific, Waltham, MA, USA); TYRP-1, Covance #SIG-38150 (Covance Inc., Princeton, NJ, USA), MITF, Abcam #ab80651 (Abcam plc, Cambridge, UK). The number of positive cells within the epidermis was counted and the value was normalized upon the length of the basal lamina, thus obtaining a score.

### 4.8. Nrf2 Activation in Skin Tissue after UVA-Irradiation

On an abdominoplasty coming from a 33-year-old Caucasian woman with Fitzpatrick phototype II, skin explants of an average diameter of 11 mm were prepared after obtaining written informed consent. The explants were kept in culture medium at 37 °C in a humid, 5% CO_2_ atmosphere. The peptide was applied topically and spread using a small spatula on day 0 (D0), D1, D2, and D3. The control explants did not receive treatment. On day 3, explants were irradiated with UVA (13.5 J/cm^2^ equal to 3 minimal erythemal doses (MED)) using a Vilbert Lourmat solar simulator RMX 3W. For the UV irradiation, the explants were transferred into 1 mL of HBSS (Hank’s buffered salt solution) medium. The non-irradiated batches were kept in 1 mL of HBSS medium. After the UV exposure, all the explants were put in fresh Bio-EC’s explant medium (BEM medium). The products were applied on the explants 3 h before and just after UVA irradiation. Twenty-four hours after UVA irradiation, the tissues were harvested and processed for histology. After fixation for 24 h in buffered formalin, the samples were dehydrated and impregnated in paraffin using a Leica TP 1010 dehydration automat. The samples were embedded using a Leica EG 1160 embedding station. Sections 5 µm thick were made using a Leica RM 2125 Minot-type microtome, and the sections were mounted on Superfrost^®^ histological glass slides (Thermo Fisher Scientific, Waltham, MA, USA). The frozen samples were cut into 7 µm thick sections using a Leica CM 3050 cryostat. Sections were then mounted on Superfrost^®^ plus silanized glass slides. The microscopical observations were realized using a Leica DMLB (Leica Microsystems, Mannheim, Germany) or Olympus BX43 microscope (Olympus Life Science, Wallisellen, Switzerland). Pictures were digitized with a numeric DP72 Olympus camera with CellD storing software. Nrf2 immunostaining was realized on sections from formol-fixed paraffin-embedded skin explants with a monoclonal anti-Nrf2 (phospho S40) antibody (Abcam, # ab76026, clone EP1809Y) diluted at 1:1200 in PBS, BSA 0.3%, Tween20 0.05% for 1 h at room temperature using a Vectastain Kit Vector amplifier system avidin/biotin, and revealed by VIP (Vector laboratories, Ref. SK-4600). The immunostaining was performed using an automated slide-processing system (Dako, AutostainerPlus).

## 5. Patents

DSM Nutritional Products is the owner of the patent application WO-2018/065345 MELANOCORTIN-1-RECEPTOR AGONIST describing the peptides mentioned in this manuscript.

## Figures and Tables

**Table 1:** MC1R activation assay results.

Entry	AA_b_	AA_a_	R^1^	R^2^	R^3^	EC_50_ (nM)
1	Arg	*L*-Trp	H	H	H	0.041
2	Arg	*L*-NaphAla	H	H	H	0.11
3	Arg	*D*-NaphAla	H	H	H	0.25
4	Arg	*D*-Trp	Propyl	Propyl	H	0.42
5	Arg	*L*-Trp	Propyl	Propyl	H	0.88
6	Arg	*D*-Phe	H	H	H	1.79
7	Arg	Gly	H	H	CH_2_-3-Indolyl	4.4
8	Dab	*L*-Trp	H	H	H	7.51

**Table 2:** EC_50_ values of peptide 4 on all GPCRs tested. EC_50_ values were determined using eight concentrations in a range from 0.01 to 100,000 nM. EC_50_ on MC1R is from Table 1 and shown for comparison. For abbreviations and explanations on the various receptors see Discussion.

Peptide Entry	Receptor	EC_50_ [nM] (Activation)
4	MC1R	0.42
4	MC2R	> 100,000
4	MC3R	877
4	MC4R	136
4	MC5R	> 100,000
4	MT1R	> 100,000
4	MT2R	> 100,000
4	OP1R	> 100,000
4	OP2R	> 100,000
4	OP3R	> 100,000
4	MCH1R	> 100,000
4	MCH2R	> 100,000
4	ETBR	> 100,000
4	5-HT_2A_R	> 100,000

## Supplementary Materials

Supplementary materials can be found at https://www.mdpi.com/1422-0067/20/24/6143/s1.
