# Supplementary material for: Discovery of a Highly Selective MC1R Agonists Pentapeptide to Be Used as a Skin Pigmentation Enhancer and with Potential Anti-Aging Properties
Source: Int J Mol Sci. 2019 Dec 5;20(24):6143. doi: 10.3390/ijms20246143 (PMC6940745; doi:10.3390/ijms20246143)
Supplement: Supplementary file 1 [file ijms-20-06143-s001.pdf]

Supplementary Materials

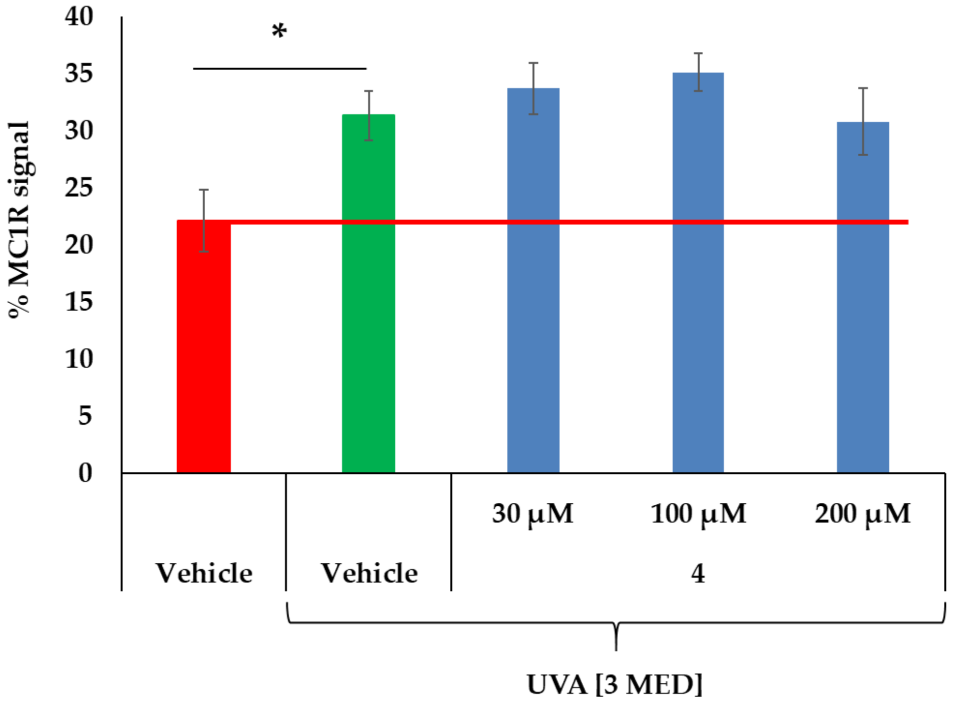

Figure S1. MC1R protein expression. UVA induced MC1R expression in skin ex vivo [1]. However, peptide 4 did not further modulate MC1R expression compared to the UVA treated vehicle control under these conditions. Experiment was done the same way as detection of Nrf2 (see Material and Methods section in main document, section 4.8.). The antibody used was a polyclonal anti-MC1R antibody (Novus Bilogicals, Ref.: NLS1040) diluted at 1:100 in PBS-BSA 0.3%-Tween 20 0.05% for 1 h at room temperature and revealed by AlexaFluor488 (Lifetechnologies, Ref. A11008). Error bars represent standard error of the mean. * $p < 0.05$ vs vehicle by unpaired Student's T-test.

Reference

1. Schiller, M.; Brzoska, T.; Bohm, M.; Metze, D.; Scholzen, T.E.; Rougier, A.; Luger, T.A. Solar-simulated ultraviolet radiation-induced upregulation of the melanocortin-1 receptor, proopiomelanocortin, and alpha-melanocyte-stimulating hormone in human epidermis in vivo. *J. Invest. Dermatol.* **2004**, *122*, 468–476.

Synthesis Strategy

Unsubstituted amides were prepared on rink linker amide resin using solid phase synthesis approach. After simultaneous cleavage of the side chain protecting groups as well as the attachment to the resin, the crude peptide is purified by preparative HPLC.

Substituted amides were prepared on 2-chloro trityl resin side as chain protected peptides with free acid and coupled with the corresponding amine to give the side chain protected test item in solution, which is deprotected and thoroughly purified by HPLC.

Typical procedure for the preparation of the free amides

Approximately 2.5 g Fmoc-ramage-resin (loading approx. 0.5 mmol/g) is placed in a peptide synthesizer reaction tube and the sequence is assembled on a peptide synthesizer. 1.25 eq of the respective Fmoc-amino acids (side-chain functional groups may be Boc/Pbf/Trt) protected if present) are coupled to the growing peptide chain with 1.25 eq TBTU and 3 eq DIPEA. Fmoc protection group

is removed with 4 methyl piperidine. The *N*-terminal Bz group is attached using 1.1 eq benzoyl chloride with a 5-fold excess of DIPEA.

The fully assembled peptide is cleaved from the resin with 25.8 ml of a TFA/TIPS/DCM = 22.5/0.8/2.5 mixture (v/v). The crude peptide is precipitated by adding the solution to a 200ml of IPE/Hexan = 1/1 (v/v). The precipitate is directly purified by preparative HPLC resulting in the yields as given in table 2.

Table SI: Free amide peptides.

Entry	Sequence	Amount, yield
1	Bz-Gly-His-D-Phe-Arg-Trp-NH_2 *2TFA	196 mg (19%)
2	Bz-Gly-His-D-Phe-Arg-L-2-NaphAla- NH_2 *2TFA	413mg (39%)
3	Bz-Gly-His-D-Phe-Arg-D-2-NaphAla-NH_2 *2TFA	351mg (32%)
6	Bz-Gly-His-D-Phe-Arg-D-Phe-NH_2 *2TFA	218 mg (22%)
7	Bz-Gly-His-D-Phe-Arg-(N-IndEt)Gly-NH_2 *2TFA	135 mg, (13%)
8	Bz-Gly-His-D-Phe-Dab-Trp- NH_2 *2TFA	170 mg (14%)

Typical procedure for the preparation of the substituted amides

Approximately 2 g 2-chloro-trityl-resin (loaded with the fist amino acid approx. 0.5 mmol/g) is placed in a peptide synthesizer reaction tube and the sequence is assembled on a peptide synthesizer. 1.25 eq of the respective Fmoc-amino acids (side-chain functional groups are Boc/Pbf/Trt) protected if present) are coupled to the growing peptide chain with 1.25 eq TBTU and 3 eq DIPEA. Fmoc protection group is removed with 4 methyl piperidine. The *N*-terminal Bz group is attached using 1.1 eq benzoyl chloride with a 5-fold excess of DIPEA.

The fully assembled peptide is cleaved from the resin with three times 20 ml of DCM containing 0.1% TFA. The combined DCM portions are combined in a separatory funnel and washed neutral. Organic phase is dried over Na_2SO_4 and all volatile compounds removed in vacuum. Crude peptide is carefully coupled using 3 eq of 2,4,6-trimethylpyridine 1 eq of TPTU and 1.1 eq amine at 0°C. Regular aqueous workup ($NaHCO_3$, $KHSO_4$, NaCl) is followed by removal of all side chain protecting groups with TFA/TIPS/DCM = 22.5/0.8/2.5 mixture (v/v) and precipitation by adding the solution to IPE/Hexan = 1/1 (v/v). The precipitate is directly purified by preparative HPLC resulting in the yields as given in table 3.

Table SII: Substituted amide peptides.

Entry	Sequence	Amount, yield
4	Bz-Gly-His-D-Phe-Arg-D-Trp-N(Propyl)$_2$ *2TFA	131 mg (12%)
5	Bz-Gly-His-D-Phe-Arg-Trp-N(Propyl)$_2$ *2TFA	196 mg (36%)

Characterisation of 1

^{1}H NMR (600 MHz, DMSO-d_6) δ ppm 1.22 (quin, J=7.48 Hz, 2 H), 1.32 - 1.43 (m, 1 H), 1.53 - 1.66 (m, 1 H), 2.77 (dd, J=15.34, 8.94 Hz, 1 H), 2.82 (dd, J=13.36, 9.03 Hz, 1 H), 2.91 - 3.03 (m, 5 H), 3.14 (dd, J=14.59, 5.18 Hz, 1 H), 3.80 - 3.93 (m, 2 H), 4.18 (td, J=8.23, 5.18 Hz, 1 H), 4.44 (td, J=8.28, 5.46 Hz, 1 H), 4.59 (dd, J=15.06, 7.72 Hz, 1 H), 4.63 (td, J=8.61, 4.80 Hz, 1 H), 6.65 – 7.50 (br. s., 4H), 6.95 (ddd, J=7.91, 7.06, 1.04 Hz, 1 H), 7.03 (td, J=7.53, 1.13 Hz, 1 H), 7.06 - 7.08 (m, 2 H), 7.13 (d, J=2.26 Hz, 1 H), 7.15 - 7.18 (m, 1 H), 7.20 - 7.25 (m, 4 H), 7.29 - 7.33 (m, 2 H), 7.44 - 7.49 (m, 2 H), 7.51 - 7.56 (m, 2 H), 7.58 (d, J=8.09 Hz, 1 H), 7.85 - 7.89 (m, 2 H), 7.97 (d, J=8.09 Hz, 1 H), 8.25 (d, J=8.28 Hz, 1 H), 8.32 (d, J=7.72 Hz, 1 H), 8.38 (d, J=7.53 Hz, 1 H), 8.79 (t, J=5.74 Hz, 1 H), 8.95 (d, J=1.32 Hz, 1 H), 10.77 (d, J=2.07 Hz, 1 H), 14.19 (br. s., 2 H).

^{13}C NMR (151 MHz, DMSO-d_6) δ ppm 24.73, 27.06, 27.59, 28.92, 37.89, 40.04, 40.37, 42.81, 51.36, 52.35, 53.36, 54.36, 110.09, 111.27, 116.62, 116.70 (q, *J*=297 Hz), 118.24, 118.42, 120.84, 123.50, 126.39, 127.26, 127.33, 128.06, 128.31, 129.11, 129.22, 131.46, **133.70**, 133.73, 136.00, 137.24, 156.68, 158.34 (q, *J*=32.74 Hz), 166.78, 169.25, 169.86, 170.95, 171.04, 173.37.

Chemical Formula: C41H48N12O6 *2C2HF3O2

Exact Mass: 804.38

Exact Mass: $[M+2H]^+$ = 403.1990   calc. 403.1983

UPLC: 2_30min_100_HSS_T3_02TFA R_t = 6.49 min

Characterisation of 2

^1H NMR (600 MHz, DMSO-d_6) δ ppm 1.19 (quin, *J*=7.72 Hz, 2 H), 1.30 - 1.40 (m, 1 H), 1.49 - 1.58 (m, 1 H), 2.73 - 2.84 (m, 2 H), 2.91 (q, *J*=7.15 Hz, 2 H), 2.93 - 2.98 (m, 1 H), 3.01 (dd, *J*=13.83, 9.13 Hz, 1 H), 3.20 (dd, *J*=13.93, 5.08 Hz, 1 H), 3.79 - 3.84 (m, 1 H), 3.86 - 3.92 (m, 1 H), 4.14 (td, *J*=8.23, 5.36 Hz, 1 H), 4.51 (td, *J*=8.66, 5.27 Hz, 1 H), 4.54 - 4.60 (m, 1 H), 4.63 (td, *J*=8.61, 4.80 Hz, 1 H), 6.70 – 7.50 (br. s, 5H), 7.08 (s, 1 H), 7.12 (s, 1 H), 7.15 - 7.18 (m, 1 H), 7.19 - 7.26 (m, 4 H), 7.34 (s, 1 H), 7.41 (dd, *J*=8.81, 1.30 Hz, 1 H), 7.45 - 7.49 (m, 5 H), 7.53 - 7.56 (m, 1 H), 7.70 (s, 1 H), 7.76 - 7.82 (m, 2 H), 7.84 (dd, *J*=7.62, 1.04 Hz, 1 H), 7.87 (dd, *J*=8.28, 1.32 Hz, 2 H), 8.03 (d, *J*=8.09 Hz, 1 H), 8.23 (d, *J*=8.28 Hz, 1 H), 8.31 (d, *J*=7.72 Hz, 1 H), 8.37 (d, *J*=7.53 Hz, 1 H), 8.80 (t, *J*=5.74 Hz, 1 H), 8.95 (d, *J*=1.13 Hz, 1 H), 13.87 - 14.47 (m, 2 H).

^{13}C NMR (151 MHz, DMSO-d_6) δ ppm 24.68, 27.04, 28.82, 37.54, 37.87, 40.05, 40.31, 42.86, 51.36, 52.44, 53.90, 54.36, 116.51 (q, *J*=296 Hz), 116.61, 125.40, 125.93, 126.39, 127.33, 127.36, 127.43, 127.48, 127.75, 128.06, 128.31, 129.10, 129.20, 131.48, 131.80, 132.90, 133.71 (two signals: Cq(Bz)+ NH-CH=N (His)), 135.58, 137.21, 156.63, 158.23 (q, *J*=33.5 Hz), 166.80, 169.22, 169.84, 171.00, 171.10, 172.81.

Chemical Formula: C43H49N11O6 *2C2HF3O2

Exact Mass: 815.39

Exact Mass: $[M+2H]^+$ = 408.7007   calc 408.7006

UPLC: 2_30min_100_HSS_T3_02TFA R_t = 7.60 min

Characterisation of 3

^1H NMR (600 MHz, DMSO-d_6) δ ppm 0.89 - 1.02 (m, 2 H), 1.12 - 1.20 (m, 1 H), 1.31 - 1.39 (m, 1 H), 2.64 - 2.78 (m, 4 H), 2.86 - 2.96 (m, 3 H), 3.22 (dd, *J*=13.55, 4.33 Hz, 1 H), 3.79 - 3.89 (m, 3 H), 4.20 - 4.25 (m, 1 H), 4.55 (td, *J*=9.46, 4.61 Hz, 1 H), 4.59 - 4.64 (m, 2 H), 6.70-7.60 (br.s., 4H), 7.00 (s, 1 H), 7.12 - 7.16 (m, 1 H), 7.17 (s, 1 H), 7.19 - 7.23 (m, 4 H), 7.37 (t, *J*=5.55 Hz, 1 H), 7.41 (dd, *J*=8.47, 1.69 Hz, 1 H), 7.51 - 7.55 (m, 2 H), 7.69 (s, 1 H), 7.77 - 7.81 (m, 2 H), 7.83 - 7.87 (m, 3 H) 8.21 - 8.26 (m, 2 H), 8.33 (d, *J*=8.47 Hz, 2 H), 8.31 - 8.35 (m, 2 H), 8.77 (t, *J*=5.83 Hz, 1 H), 8.93 (d, *J*=1.13 Hz, 1 H), 14.12 (br. s., 2 H).

^{13}C NMR (151 MHz, DMSO-d_6) δ ppm 24.36, 27.10, 28.98, 37.96, 38.17, 40.11, 42.75, 51.31, 51.94, 53.82, 53.94, 116.52, 117.03 (q, *J*=296 Hz), 125.36, 125.92, 126.33, 127.30, 127.43, 127.52, 127.78, 128.00, 128.29, 129.07, 129.21, 131.44, 131.76, 132.86, **133.65**, 133.74, 135.72, 37.39, 156.60, 158.21 (q, *J*=31.8 Hz), 166.71, 169.19, 169.51, 170.70, 170.75, 172.88.

Chemical Formula: C43H49N11O6 *2C2HF3O2

Exact Mass: 815.39

Exact Mass: $[M+2H]^+$ = 408.7007   calc 408.7006

UPLC: 2_30min_100_HSS_T3_02TFA R_t = 7.62 min

Characterisation of 4

^{1}H NMR (600 MHz, DMSO-d_6) δ ppm 0.58 (t, J=7.34 Hz, 3 H), 0.70 (t, J=7.43 Hz, 3 H), 1.11 - 1.26 (m, 3 H), 1.28 - 1.43 (m, 4 H), 1.46 - 1.54 (m, 1 H), 2.69 - 2.78 (m, 2 H), 2.85 - 3.00 (m, 8 H), 3.07 (dd, J=14.21, 7.43 Hz, 1 H), 3.27 (ddd, J=13.13, 8.61, 6.31 Hz, 1 H), 3.82 - 3.90 (m, 2 H), 4.36 - 4.42 (m, 1 H), 4.60 (td, J=8.38, 4.89 Hz, 1 H), 4.64 - 4.69 (m, 1 H), 4.90 - 4.96 (m, 1 H), 6.65 – 7.50 (br. s, 4 H), 6.96 - 6.99 (m, 2 H), 7.05 (td, J=7.53, 1.13 Hz, 1 H), 7.09 (d, J=2.45 Hz, 1 H), 7.13 - 7.17 (m, 1 H), 7.21 - 7.24 (m, 4 H), 7.32 (d, J=8.09 Hz, 1 H), 7.43 - 7.47 (m, 2 H), 7.49 - 7.55 (m, 2 H), 7.57 (d, J=8.09 Hz, 1 H), 7.85 (dd, J=8.38, 1.22 Hz, 2 H), 8.21 (d, J=8.28 Hz, 1 H), 8.30 (d, J=8.28 Hz, 1 H), 8.34 (d, J=8.47 Hz, 1 H), 8.41 (d, J=8.47 Hz, 1 H), 8.76 (t, J=5.74 Hz, 1 H), 8.93 (d, J=1.13 Hz, 1 H), 10.80 (d, J=2.07 Hz, 1 H), 14.13 (br. s., 2 H).

^{13}C NMR (151 MHz, DMSO-d_6) δ ppm 10.76, 11.14, 20.35, 21.80, 24.65, 27.13, 28.62, 29.66, 38.22, 40.28, 42.75, 47.19, 48.69, 49.23, 51.33, 51.58, 53.92, 109.50, 111.43, 116.50, 117.95, 118.33, 120.94, 123.87, 126.32, 127.10, 127.29, 128.00, 128.27, 129.07, 129.25, 131.43, 133.64, 133.77, 136.07, 137.47, 156.64, 158.23 (q, J=31.91 Hz), 166.69, 169.16, 169.42, 170.45, 170.58, 170.73.

Chemical Formula: C47H60N12O6 *2C2HF3O2

Exact Mass: 888.48

Exact Mass: $[M+2H]^{+}$ = 445.2459 calc. 445.2452

UPLC: 2_1m30s_100_1m_100_T3_02TFA R_t = 9.67 min

Characterisation of 5

^{1}H NMR (600 MHz, DMSO-d_6) δ ppm 0.54 (t, J=7.34 Hz, 3 H), 0.67 (t, J=7.34 Hz, 3 H), 1.01 - 1.12 (m, 1 H), 1.28 (d, J=7.34 Hz, 5 H), 1.37 - 1.48 (m, 1 H), 1.55 - 1.67 (m, 1 H), 2.72 - 2.81 (m, 2 H), 2.82 - 2.89 (m, 1 H), 2.89 - 3.05 (m, 7 H), 3.11 (dd, J=14.12, 8.28 Hz, 1 H), 3.16 (ddd, J=13.18, 8.75, 6.12 Hz, 1 H), 3.81 - 3.88 (m, 1 H), 3.89 - 3.96 (m, 1 H), 4.35 (td, J=8.19, 5.65 Hz, 1 H), 4.59 - 4.69 (m, 2 H), 4.86 - 4.96 (m, 1 H), 6.94 - 6.99 (m, 1 H), 7.04 (s, 2 H), 7.10 (d, J=2.26 Hz, 1 H), 7.13 - 7.19 (m, 1 H), 7.20 - 7.26 (m, 4 H), 7.32 (d, J=8.09 Hz, 1 H), 7.43 - 7.49 (m, 2 H), 7.50 - 7.55 (m, 1 H), 7.56 – 7.57 (m, 2 H), 7.84 - 7.88 (m, 2 H), 8.26 (d, J=8.09 Hz, 1 H), 8.31 (d, J=8.28 Hz, 1 H), 8.34 (d, J=8.09 Hz, 1 H), 8.42 (d, J=8.09 Hz, 1 H), 8.78 (t, J=5.83 Hz, 1 H), 8.94 (d, J=1.32 Hz, 1 H), 10.82 (d, J=1.88 Hz, 1 H), 14.17 (br. s., 2 H).

^{13}C NMR (151 MHz, DMSO-d_6) δ ppm ppm 10.70, 11.06, 20.24, 21.67, 24.70, 27.18, 28.42, 29.40, 38.09, 40.41, 42.72, 47.23, 48.57, 49.26, 51.35, 51.63, 54.10, 109.47, 111.43, 116.54, 116.96 (q, *J*=299 Hz), 117.99, 118.33, 120.94, 123.76, 126.35, 127.15, 127.31, 128.02, 128.28, 129.08, 129.25, 131.41, 133.64, 133.78, 136.05, 137.39, 156.68, 158.25 (q, *J*=31.9 Hz), 166.67, 169.19, 169.57, 170.56, 170.61, 170.78.

Chemical Formula: C47H60N12O6 *2C2HF3O2

Exact Mass: 888.48

Exact Mass: $[M+2H]^{+}$ = 445.2461 calc. 445.2361

UPLC: 2_30min_100_HSS_T3_02TFA R_t =9.82 min

Characterisation of 6

^{1}H NMR (600 MHz, DMSO-d_6) δ ppm 0.92 - 1.10 (m, 2 H), 1.20 - 1.29 (m, 1 H), 1.33 - 1.42 (m, 1 H), 2.68 - 2.78 (m, 3 H), 2.84 - 2.92 (m, 3 H), 2.96 (dd, J=13.65, 5.18 Hz, 1 H), 3.07 (dd, J=13.65, 4.23 Hz, 1 H), 3.80 - 3.91 (m, 2 H), 4.19 - 4.25 (m, 1 H), 4.45 (ddd, J=10.45, 8.85, 4.23 Hz, 1 H), 4.62 (s, 2 H), 6.83 – 7.50 (m, 4 H), 7.00 (s, 1 H), 7.13 - 7.19 (m, 3 H), 7.21 - 7.26 (m, 8 H), 7.45 - 7.51 (m, 4 H), 7.53 - 7.58 (m,

1 H), 7.87 (dd, J=8.38, 1.41 Hz, 2 H), 8.23 (d, J=8.28 Hz, 1 H), 8.27 (d, J=7.72 Hz, 1 H), 8.31 (d, J=8.85 Hz, 1 H), 8.34 (d, J=8.47 Hz, 1 H), 8.79 (t, J=5.83 Hz, 1 H), 8.93 (d, J=1.13 Hz, 1 H), 14.15 (br. s., 2 H).
^{13}C NMR (151 MHz, DMSO-*d*$_6$) δ ppm 24.39, 27.13, 28.99, 37.62, 38.23, 40.05, 40.26, 42.73, 51.32, 52.08, 53.84, 53.86, 116.52, 117.10 (q, J = 299Hz), 126.21, 126.33, 127.31, 127.98, 128.01, 128.30, 129.09, 129.15, 129.22, 131.44, 133.65, 133.77, 137.43, 138.01, 156.64, 158.29 (q, J = 31.5 Hz), 166.70, 169.18, 169.46, 170.72, 170.76, 173.00.
Chemical Formula: C39H47N11O6 *2C2HF3O2
Exact Mass: 765.37
Exact Mass: [M+2H]$^+$ = 383.6933 calc. 383.6928
Exact Mass: [M+H]$^+$ = 766.3776 calc. 766.3770
UPLC: 2_30min_100_HSS_T3_02TFA R$_t$ = 6.00min

Characterisation of 7

^{1}H NMR (600 MHz, DMSO-*d*$_6$) δ ppm 1.16 - 1.26 (m, 0.6 H, min), 1.28 - 1.42 (m, 1.4 H), 1.43 - 1.55 (m, 1.6 H), 1.65 - 1.72 (m, 0.4 H, min), 2.65 - 2.90 (m, 4 H), 2.91 - 3.10 (m, 4 H), 3.42 - 3.53 (m, 0.8 H, min), 3.57 - 3.68 (m, 1.2 H, Maj), 3.74 (d, J=16.38 Hz, 0.6 H, Maj), 3.79 - 3.90 (m, 2 H), 3.92 (d, J=16.60 Hz, 0.4 H, min), 4.05 (d, J=16.19 Hz, 0.6 H, Maj), 4.25 (d, J=17.50 Hz, 0.4 H, min), 4.51 - 4.59 (m, 0.4 H, min), 4.62 (td, J=8.52, 4.99 Hz, 1 H), 4.68 - 4.77 (m, 1.6 H), 6.50 – 7.60 (m, 4 H), 6.94 - 6.98 (m, 1.6 H), 6.99 - 7.02 (m, 1H), 7.04 - 7.09 (m, 1 H), 7.11 (d, J=2.26 Hz, 1 H), 7.14 - 7.18 (m, 1 H), 7.18 (d, J=2.26 Hz, 1 H), 7.21 - 7.28 (m, 4.6 H), 7.32 - 7.36 (m, 1 H), 7.43 - 7.48 (m, 2 H), 7.50 - 7.55 (m, 1.6 H), 7.56 (t, J=5.74 Hz, 0.4 H), 7.58 - 7.62 (m, 1 H), 7.83 - 7.89 (m, 2 H), 8.20 (d, J=8.28 Hz, 0.6 H, Maj), 8.23 (d, J=8.28 Hz, 0.4 H, min), 8.36 (d, J=8.85 Hz, 1 H), 8.45 - 8.53 (m, 1 H), 8.71 - 8.80 (m, 1 H), 8.93 (d, J=1.13 Hz, 0.6 H, Maj), 8.94 (d, J=1.13 Hz, 0.4 H, min), 10.79 (d, J=1.88 Hz, 0.4 H, min), 10.87 (d, J=1.88 Hz, 0.6 H, Maj) 14.14 (br. s., 2 H).
^{13}C NMR (151 MHz, DMSO-*d*$_6$) δ ppm 22.91 min, 24.31 Maj, 24.77 Maj, 27.21, 29.06 min, 29.16 Maj, 38.43 min, 38.57 Maj, 40.56 Maj, 40.60 min, 42.70, 48.15 Maj, 48.26 min, 48.29 min, 48.58 Maj, 49.60 Maj, 50.23 min, 51.29 Maj, 51.34 min, 53.65 Maj, 53.72 min, 110.77 Maj, 111.26 min, 111.43 min, 111.45 Maj, 116.48, 116.49 (q, *J*=296 Hz), 118.15 min, 118.24 Maj, 118.25 min, 118.40 Maj, 120.96 min, 121.02 Maj, 122.68 min, 123.25 Maj, 126.33, 127.02 Maj, 127.09 min, 127.30 min, 127.32 Maj, 128.00, 128.29, 129.08, 129.28, 131.41, 136.64, 133.78, 136.18 Maj, 136.22 min, 137.48 Maj, 137.52 min, 156.66 Maj, 156.68 min, 158.22 (q, *J*=31.36 Hz), 166.67 Maj, 166.69 min, 169.12 Maj, 169.20 min, 169.35 Maj, 169.40 min, 170.17 Maj, 170.36 min, 170.51 min, 170.67 Maj, 171.23 min, 171.40 Maj.
Chemical Formula: C42H50N12O6 *2C2HF3O2
Exact Mass: 818.40
Exact Mass: [M+2H]$^+$ = 410.2066 calc. 410.2061
UPLC: 2_30min_100_HSS_T3_02TFA R$_t$ = 7.32 min

Characterisation of 8

^{1}H NM^{1}H NMR (600 MHz, DMSO-*d*$_6$) δ ppm 1.70 - 1.79 (m, 1 H), 1.84 - 1.92 (m, 1 H), 2.60 - 2.71 (m, 2 H), 2.74 (dd, J=15.34, 8.94 Hz, 1 H), 2.79 (dd, J=13.55, 9.60 Hz, 1 H), 2.91 (dd, J=15.25, 4.71 Hz, 1 H), 2.95 - 3.02 (m, 2 H), 3.14 (dd, J=14.78, 5.18 Hz, 1 H), 3.78 - 3.94 (m, 2 H), 4.33 - 4.41 (m, 1 H), 4.46 (td, J=8.05, 5.55 Hz, 1 H), 4.54 - 4.68 (m, 2 H), 6.96 (td, J=7.43, 0.94 Hz, 1 H), 7.01 - 7.05 (m, 2 H), 7.09 (s, 1 H), 7.14 (d, J=2.26 Hz, 1 H), 7.15 - 7.19 (m, 1 H), 7.22 - 7.26 (m, 4 H), 7.31 (d, J=8.09 Hz, 1 H), 7.43 (s, 1 H), 7.45 - 7.50 (m, 2 H), 7.53 - 7.57 (m, 1 H), 7.60 (d, J=7.91 Hz, 1 H), 7.75 (br. s., 3 H), 7.85 - 7.88 (m, 2

H), 8.09 (d, J=7.72 Hz, 1 H), 8.24 (d, J=8.28 Hz, 1 H), 8.40 (d, J=7.91 Hz, 1 H), 8.46 (d, J=7.91 Hz, 1 H), 8.78 (t, J=5.84 Hz, 1 H), 8.94 (d, J=1.13 Hz, 1 H), 10.79 (d, J=1.88 Hz, 1 H), 14.20 (br. s., 2 H).

^{13}C NMR (151 MHz, DMSO-d_6) δ ppm 27.09, 27.69, 30.15, 35.85, 37.93,
42.78, 50.11, 51.33, 53.39, 54.19, 109.90, 111.29, 116.49 (q, *J*=296 Hz), 116.54, 118.27, 118.45, 120.89, 123.54, 126.45, 127.26, 127.32, 128.08, 128.31, 129.07, 129.22, 131.46, 133.69, 133.75, 136.02, 137.35, 158.22 (q, *J*=31.36 Hz), 166.77, 169.22, 170.11, 173.21.

Chemical Formula: C39H44N10O6 *2C2HF3O2

Exact Mass: 748.34

Exact Mass: $[M+2H]^+$ = 375.1805 calc. 375.1795

Exact Mass: $[M+H]^+$ = 749.3526 calc. 749.3518

UPLC: 2_30min_100_HSS_T3_02TFA R_t = 6.36 min
